# S100a8/A9 proteins: critical regulators of inflammation in cardiovascular diseases

**DOI:** 10.3389/fcvm.2024.1394137

**Published:** 2024-08-08

**Authors:** Yu Sun, Han Xu, Weihan Gao, Jinlan Deng, Xiayinan Song, Jie Li, Xijian Liu

**Affiliations:** ^1^ College of Traditional Chinese Medicine, Shandong University of Traditional Chinese Medicine, Jinan, China; ^2^ Innovation Research Institute of Traditional Chinese Medicine, Shandong University of Traditional Chinese Medicine, Jinan, China

**Keywords:** cardiovascular disease, S100A8/A9 protein, inflammation, neutrophil, biomarker

## Abstract

Neutrophil hyperexpression is recognized as a key prognostic factor for inflammation and is closely related to the emergence of a wide range of cardiovascular disorders. In recent years, S100 calcium binding protein A8/A9 (S100A8/A9) derived from neutrophils has attracted increasing attention as an important warning protein for cardiovascular disease. This article evaluates the utility of S100A8/A9 protein as a biomarker and therapeutic target for diagnosing cardiovascular diseases, considering its structural features, fundamental biological properties, and its multifaceted influence on cardiovascular conditions including atherosclerosis, myocardial infarction, myocardial ischemia/reperfusion injury, and heart failure.

## Introduction

In recent years, the incidence rate and mortality rate of cardiovascular diseases have been increasing year by year (1). Despite the ongoing enhancements in diagnostic and treatment technologies, several shortcomings remain, such as extended diagnostic timeframes, suboptimal sensitivity and specificity of existing diagnostic markers, and, most notably, a dearth of targeted medications that are both safe and effective for particular cardiovascular conditions. Thus, the pursuit of new markers and therapeutic targets is essential for the prevention and treatment of cardiovascular diseases (2). The impact of immune system-mediated myocardial injury and the associated development of cardiovascular disease is a burgeoning area of interest in the study of disease mechanisms. Evidence has established that the innate immune response is stimulated by damage associated molecular patterns (DAMPs), leading to inflammatory processes. S100A8/A9, a component of the S100 calponin family of DAMPs, forms the S100A8/A9 heterodimer, also known as MRP8/14, and is consistently expressed (3). S100A8/A9 importance in cardiovascular diseases is underscored by its association with the extent of atherosclerosis in coronary and carotid arteries, plaque vulnerability, and its role in myocardial infarction and myocardial ischemia/reperfusion injury. Consequently, S100A8/A9 may be a promising biomarker and therapeutic target for cardiovascular conditions. The development and clinical trial approval of S100A8/A9 receptor blockers further underscore its potential (4). This review explores S100A8/A9's utility as a diagnostic tool for cardiovascular system effects and as a marker for cardiovascular events, providing a theoretical basis for the clinical treatment of cardiovascular diseases.

### Overview of the S100 protein family

The S100 protein family, initially identified by Moore in 1965, comprises small molecular weight proteins (10–12 kDa) known for their solubility in a 100% neutral saturated ammonium sulfate solution and their role as calcium-regulatory proteins (5).This family now encompasses 25 members, including S100A1-16, S100G, and S100B, all of which share a significant degree of sequence and structural similarity and possess a characteristic calcium-binding motif (6). Each protein consists of two helix-loop-helix EF-hand domains surrounded by conserved hydrophobic regions at the N- and C-termini, connected by a central hinge. The N-terminus typically contains a 14-amino acid loop with low calcium affinity, whereas the C-terminus includes a high-affinity calcium-binding loop of 12 amino acids. This structural configuration facilitates a conformational change upon calcium binding, exposing hydrophobic domains that interact with various target receptors and proteins, a feature essential for the functional diversity of the S100 proteins (7). Exclusively found in vertebrates, S100 proteins are highly conserved across species. Each member of the S100 family exhibits unique tissue-specific expression patterns, determined by their respective genes (8).

Unlike calmodulin, which regulates intracellular biological activity by binding Ca^2+^, S100 proteins can modulate both intracellular and extracellular functions, participating in autocrine and paracrine signaling pathways (9). These proteins influence a wide array of physiological processes by interacting with cell membrane receptors such as Receptor of Advanced Glycation Endproducts (RAGE) and G Protein-Coupled Receptors (GPCRs), impacting cell proliferation, differentiation, migration, invasiveness, inflammatory responses, oxidative stress, calcium homeostasis, apoptosis, glycogen phosphorylation, and macrophage clustering (10).

### Structure, origin and expression of S100a8/A9

**Figure 1:**
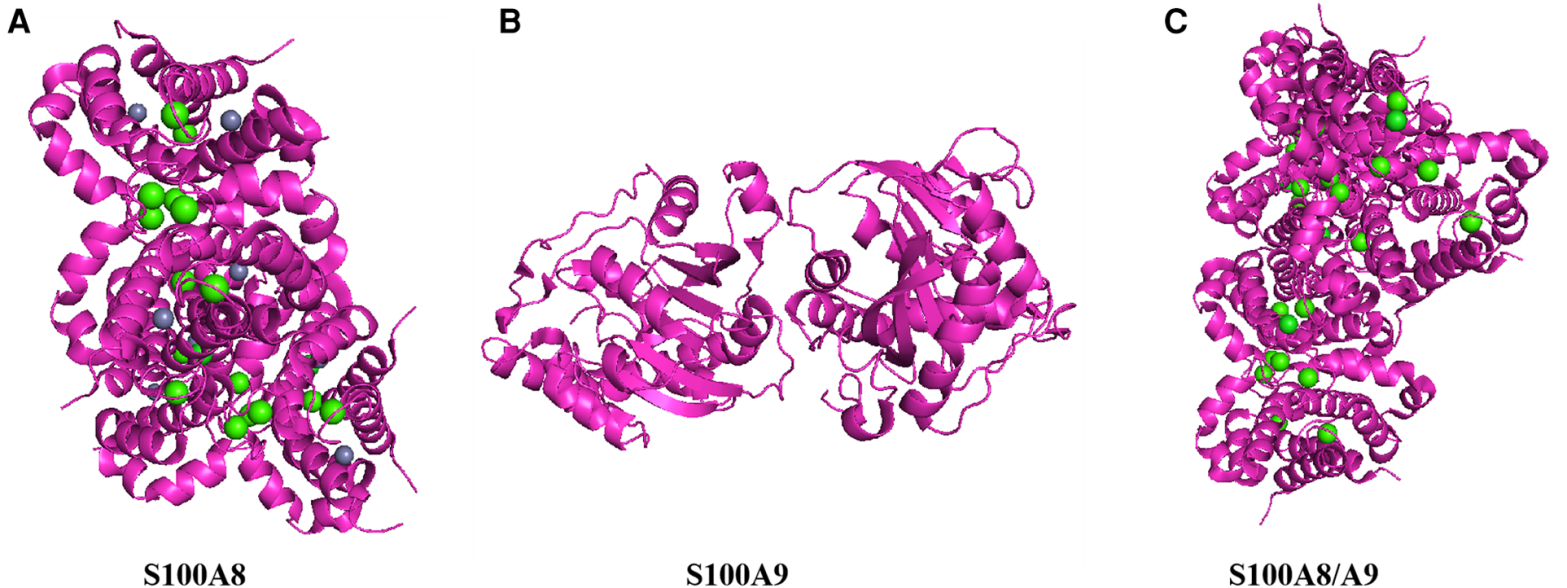
Structure of S100A8/A9. This figure shows the three-dimensional structure of S100A8/A9 heterodimer protein, demonstrating its complex folding and arrangement of key functional sites. (**A**) Color representation: Proteins are represented in magenta, displaying their alpha helix and secondary structure. Green sphere: represents the calcium ions (Ca^2+^) that bind to proteins, indicating that calcium binding sites are crucial for protein function. Gray sphere: may represent zinc ions (Zn^2+^) or other metal ions, which can stabilize protein structure and are important for its biological activity. (**B**) top-down perspective: This view provides a horizontal cross-section of proteins, emphasizing the internal arrangement of helices and loops. Subbase interface: The interface between S100A8 and S100A9 subunits is visible, indicating how they interact to form functional heterodimers. (**C**) Vertical cross-section: Provides a side view of proteins, highlighting the arrangement and spatial arrangement of alpha helices. Binding sites: The distribution of green spheres (calcium ions) throughout the structure indicates that multiple calcium binding sites are crucial for protein activity.

S100A8/A9, similar to other members of the S100 protein family, is situated at a chromosomal region known for its variability, 1q^21^, where a distinct S100 gene cluster is formed (11). The human S100A8/A9 proteins are encoded with distinct amino acid compositions; S100A8 comprises 93 amino acids and has a molecular weight of 10.8 kDa, whereas S100A9 is made up of 113 amino acids and weighs 13.2 kDa (12). These proteins can exist as homodimers, heterodimers, and tetramers, with the homodimer being less stable, leading to a preference for the formation of S100A8/A9 heterodimer complexes, which are the predominant form of S100A8/A9 in physiological settings (13). These complexes, referred to as calprotectin, possess multiple biological characteristics (14). When Ca^2+^ concentration reaches a certain threshold, S100A8/A9 heterodimers form (S100A8/A9)_2_ tetramers, a configuration that is vital for their biological activity. Secreted mainly by immune cells such as dendritic cells, neutrophils, monocytes, and activated macrophages, these proteins participate in the pathophysiology of various inflammatory conditions by attracting leukocytes and modulating the inflammatory response to vascular injury (15) (Figure 1).

## Biological functions of S100a8/A9

### S100A8/A9 regulates phagocyte migration and exacerbates inflammation development within cells

The S100A8/A9 proteins exert their biological effects through a combination of specific expression patterns, structural alterations, distinct metal ion binding affinities, and the formation of homodimers, heterodimers, and oligomers, which are implicated in various disease states (16). Intracellular S100A8/A9 plays a pivotal role in modulating the migration of phagocytic cells, thereby facilitating the progression of inflammation. By interacting with the cytosolic components p47phox and p67phox of nicotinamide adenine dinucleotide phosphate (NADPH) oxidase, intracellular S100A8/A9 enhances the activity of NADPH oxidase, exacerbating cellular oxidative stress and contributing to pro-inflammatory effects. Phosphorylation of S100A9 regulates the p38 mitogen-activated protein kinase (MAPK) signaling pathway, leading to the formation of (S100A8/A9)_2_ tetramers (17). These tetramers promote microtubule polymerization and reorganization in a calcium-dependent manner, enhancing the migration of phagocytes towards the endothelium (18) Additionally, S100A8/A9 is selectively released during the interaction between phagocytes and activated endothelial cells, making it an active mediator in the development and progression of inflammation (19).

### S100A8/A9 exerts significant inflammatory regulation extracellularly through multiple receptors

The biological activity of S100A8/A9 in the extracellular space is mediated by their interaction with receptors such as Toll-like receptor 4(TLR4) and the RAGE. Research indicates that the binding of S100A8/A9 to TLR4 sets off a signaling cascade that leads to the activation of NF-κB, which in turn governs cellular responses including inflammation and cell cycle progression. Studies have shown that S100A8/A9 can amplify the TLR4 response to Lipopolysaccharides (LPS) in myeloid cells, possibly by indirectly activating TLR4, which results in the induction of NO production by macrophages and intensifies inflammation. On the other hand, S100A8/A9 has been implicated in dampening acute inflammatory responses by regulating the activity of inflammatory cytokines (20).

**Figure 2:**
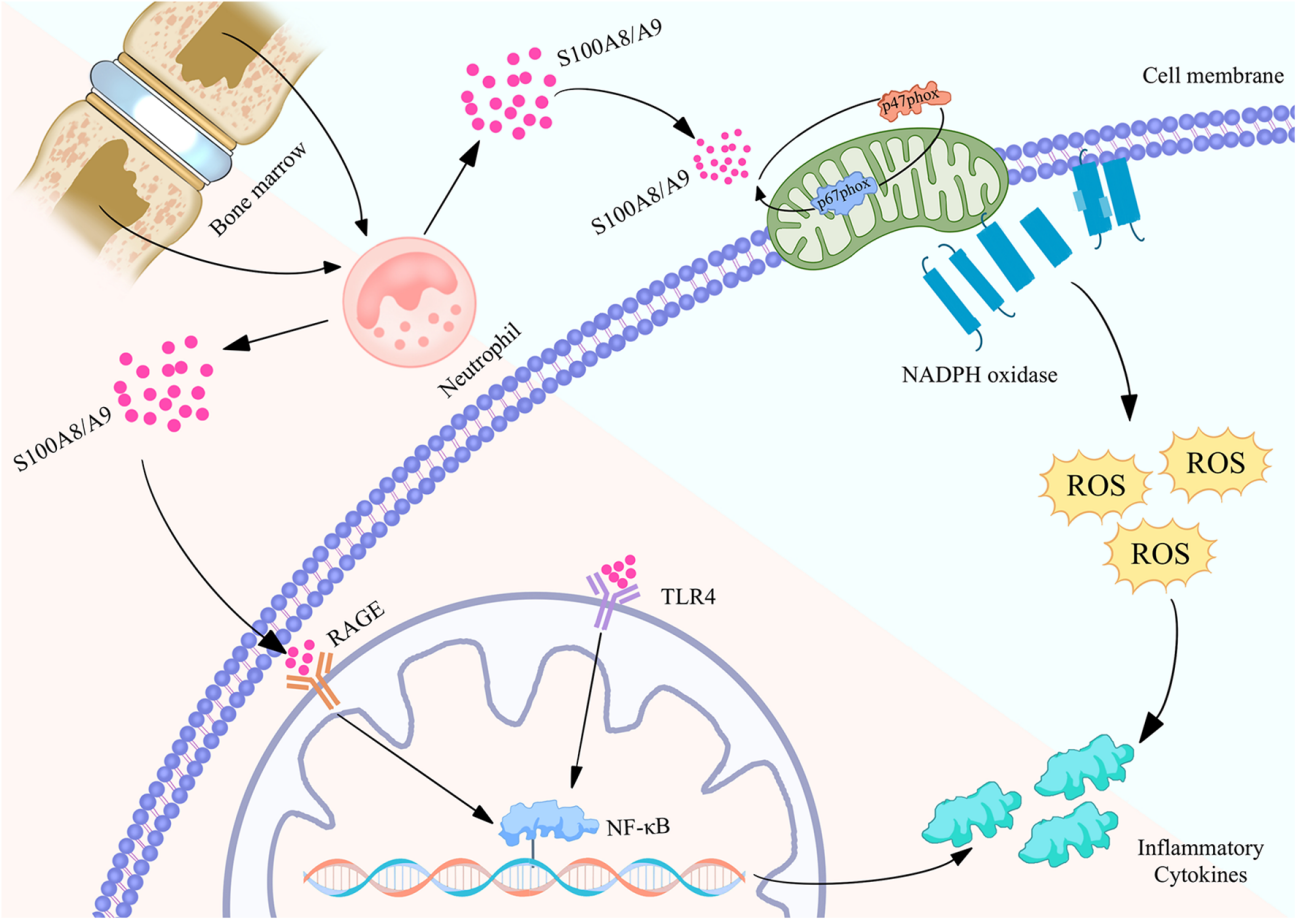
Biological function of S100A8/A9 proteins in neutrophils and their mechanism of regulating inflammatory action map. S100A8/A9 is released by neutrophils in the bone marrow and binds to RAGE and TLR4 receptors on the cell membrane, activating the NF - κB signaling pathway, thereby causing the expression of inflammatory factors. Meanwhile, S100A8/A9 can also activate NADPH oxidase, produce reactive oxygen species (ROS), and further promote inflammatory response. These pathways collectively promote the occurrence and development of inflammation, revealing the important role of S100A8/A9 in cardiovascular disease.

The protein S100A8/A9 is implicated in the inflammatory response by potentially stimulating the release of inflammatory cytokines in endothelial cells through the RAGE receptor and amplifying its activation. In vitro, S100A8/A9 has been observed to trigger cell apoptosis via both caspase-dependent and -independent pathways, contributing to endothelial injury in conditions such as vasculitis and inflammatory diseases. S100A8/A9 has been shown to suppress the proliferation and differentiation of C2C12 myoblasts and induce caspase-3-mediated apoptosis. S100A8/A9 can also decrease mitochondrial membrane potential, leading to the release of mitochondrial proteins Smac/Diablo and Omi/HtrA2, and inhibit mitochondrial fission, which in turn induces cell death by tipping the balance between pro- and anti-apoptotic factors (21). Furthermore, S100A8/A9 may induce autophagy by facilitating the relocation of the mitochondrial outer membrane protein BCL2 interacting protein 3 (BNIP3) to the mitochondria and promoting Reactive Oxygen Species (ROS) production through mitochondrial-lysosomal interactions, thereby promoting apoptosis (22). In atherosclerotic lesions, S100A8/A9 is abundantly expressed in foam cells, and its release, possibly due to the upregulation of urokinase-type plasminogen activator in macrophages, may lead to endothelial cell apoptosis. Beyond its pro-inflammatory functions, S100A8/A9 also has a regulatory role in inflammation by inhibiting dendritic cell (DC) maturation and antigen presentation, which results in a reduced T cell response and prevents an overactive adaptive immune response (23). S100A8/A9 can also augment the quantity and activity of myeloid-derived suppressor cells (MDSCs), which are known for their immunosuppressive effects in various pathological states (24). This indicates that S100A8/A9 has a multifaceted role in modulating inflammation, with different molecular biological effects in a range of cell types (Figure 2).

## Transcriptional regulation and therapeutic development of S100a8/A9 in cardiovascular diseases

### Transcriptional regulation of S100a8/A9

The transcriptional regulation of the S100A8/A9 gene is governed by multiple transcription factors, which modulate its expression in a cell-specific fashion. The S100A8/A9 gene exhibits heightened expression in human bone marrow, with mature neutrophils displaying a 40-fold increase in expression relative to monocytes (25). During inflammation in the compromised heart, specific transcription factors are implicated in the upregulation of the S100A8/A9 gene. Moreover, DNA demethylation contributes to the enhanced expression of the S100A8/A9 gene (26). This indicates a notable variation in S100A8/A9 gene expression throughout bone marrow differentiation, with its transcriptional dynamics being intricately linked to the inflammatory response mechanisms within the body. The Hoxb8 cell line is an important model system for studying the function of S100A8/A9 in myeloid cells, especially neutrophils, and can differentiate into various functional immune cell types (27). Studies have revealed that in monocytes derived from bone marrow precursor cells, S100A8/A9 mRNA levels peak during the early stages of differentiation (day 2) and subsequently decline by the later stages (days 3–5) (28). Conversely, as Hoxb8 cells differentiate into the neutrophil lineage, S100A8/A9 mRNA expression gradually increases, peaking on day 4 of culture (29). This dynamic regulation of transcription directly influences the secondary inflammatory response associated with heart failure (HF) development, thereby modulating cardiac injury and potentially decelerating the progression of cardiovascular disease.

### Protein phosphorylation and S100a8/A9 regulation

Protein phosphorylation is pivotal for the intracellular activity regulation of S100A8/A9 proteins. Schenten et al. demonstrated that phosphorylated S100A8/A9 (S100A8/A9-p) induces the expression and secretion of pro-inflammatory cytokines in HL-60 cells (30). In human neutrophils, p38-MAPK acts as the upstream kinase phosphorylating threonine 113 on S100A9, regulating the translocation of S100A8/A9 proteins from the cytoplasm to the plasma membrane, thus accelerating the inflammatory response (31). Additionally, S100A8/A9 proteins in neutrophils can undergo S-nitrosylation by nitric oxide donors, resulting in S-nitrosylated S100A8 at cysteine 41 (S100A8-SNO), which exhibits anti-inflammatory properties. S100A8-SNO inhibits eukaryotic-endothelial cell interactions in the rat mesenteric microcirculation, thereby suppressing mast cell-mediated inflammation (32). These findings suggest that S100A8/A9 transcription is regulated by multiple transcription factors under varying physiological conditions, exerting both pro-inflammatory and anti-inflammatory effects through distinct mechanisms.

### Therapeutic development of S100a8/A9 for cardiovascular diseases

The crucial role of S100A8/A9 protein in various cardiovascular diseases has led to the development of therapeutic tools and drugs targeting this protein. These therapeutic approaches aim to mitigate inflammation and oxidative stress by inhibiting the expression or function of S100A8/A9, thereby slowing the progression of cardiovascular diseases. Key therapeutic strategies include: RAGE Receptor Antagonists: Agents such as FPS-ZM1 reduce atherosclerosis and inflammatory responses by blocking the binding of S100A8/A9 to RAGE, thereby inhibiting NF-κB and MAPK pathways and reducing inflammatory cell infiltration and plaque formation (33). Anti-S100A8/A9 Antibodies: Specific antibodies that bind to S100A8/A9 prevent its interaction with receptors, inhibiting downstream inflammatory signaling pathways, reducing myocardial inflammation and apoptosis, and improving cardiac function (34). Small Molecule Inhibitors: Compounds like Paquinimod and ABR-238901 disrupt the interaction of S100A8/A9 with RAGE and TLR4, reducing plaque inflammation and instability, and reversing cardiac dysfunction in conditions such as sepsis (35). These inhibitors can bind directly to S100A8/A9 proteins or block their receptor interactions, reducing pro-inflammatory and pro-oxidative effects. Nanoparticle Delivery Systems: Utilizing nanoparticles or liposomes to deliver targeted anti-S100A8/A9 antibodies or small molecule inhibitors enhances drug concentration at the lesion site, thereby improving therapeutic efficacy (36). Gene Silencing Techniques: Techniques such as siRNA or CRISPR/Cas9 are employed to edit S100A8/A9 genes, reducing their expression and subsequent protein production. This approach decreases activation of NF-κB and MAPK pathways, reducing myocardial cell apoptosis and fibrosis (37). In summary, therapeutic tools and drugs targeting S100A8/A9 function through multiple mechanisms, including receptor antagonism, antibody therapy, small molecule inhibition, nanoparticle delivery, and gene silencing. These methods collectively work to attenuate the pro-inflammatory and pro-oxidative effects of S100A8/A9, offering significant potential in the treatment of cardiovascular diseases.

## Significance of S100a8/A9 proteins in cardiovascular disease

### S100A8/A9 regulates atherosclerotic plaque formation

S100A8/A9, as a calcium binding protein, plays an important role in the formation of atherosclerotic plaque by regulating inflammatory response and immune regulation (38, 39). Recent experimental studies have shown that S100A8/A9 activates the NF-κB signaling pathway by interacting with TLR4 and RAGE, inducing the expression of various pro-inflammatory cytokines such as tumor necrosis factor alpha TNF-α, interleukin-1β(IL-1β), and IL-6 (40). These proinflammatory factors aggravate the damage of vascular endothelial cells and promote the formation of atherosclerotic plaque (41). In addition, S100A8/A9 also plays a role in atherosclerosis by regulating oxidative stress. S100A8/A9 interacts with myeloperoxidase (MPO) to form a complex, increasing the production of reactive oxygen species (ROS) (42). ROS not only directly damages endothelial cells, but also oxidizes low-density lipoprotein (LDL), forming oxidized low-density lipoprotein (oxLDL). OxLDL is phagocytosed by macrophages to form foam cells, further promoting the formation and development of atherosclerotic plaque (43).

Other scholars explained that S100A8/A9 plays an important role in the early stage of atherosclerosis by promoting leukocyte migration and adhesion to vascular endothelial cells from the perspective of cell migration and adhesion (44). Research has shown that S100A8/A9 induces the expression of cell adhesion molecules such as Intercellular cell adhesion molecule-1(ICAM-1) and vascular cell adhesion molecule-1(VCAM-1) by binding to TLR4 and RAGE on the surface of vascular endothelial cells, enhancing the adhesion and migration of white blood cells on the arterial wall (45). At the same time, clinical studies have found that the serum S100A8/A9 level is closely related to the severity of atherosclerosis and the risk of cardiovascular events, which is expected to become a potential biomarker and therapeutic target of atherosclerosis (46). In addition, inhibiting S100A8/A9 or its signal pathway can reduce the progression of atherosclerosis, providing a new idea for the treatment of atherosclerosis (47).

In conclusion, S100A8/A9, as an important inflammatory mediator, plays a variety of roles in the occurrence and development of atherosclerosis. It promotes the formation and development of atherosclerotic plaque by regulating inflammatory response, oxidative stress, cell migration and adhesion and other mechanisms. In the future, it is expected to provide a new strategy for early diagnosis and treatment of atherosclerosis through in-depth research on the mechanism of action and clinical application value of S100A8/A9.

### S100A8/A9 regulates the inflammatory response to myocardial infarction

The pathogenesis of myocardial infarction involves a sequence of two phases: an early inflammatory stage and a subsequent repair stage. Effectively managing this transition is essential for the restoration of cardiac function and the attainment of a positive prognosis in individuals with myocardial infarction (48). S100A8/A9 contributes to the regulation of both phases of myocardial infarction, with a particular impact on the migratory and differentiative behaviors of immune cells (49). The emission of myocardial inflammatory cytokines and the state of hypoxia are hallmarks of myocardial infarction and significantly contribute to the development of ischemic damage. Duet et al. have identified that myocardial ischemia or hypoxia leads to the upregulation and secretion of S100A8 and S100A9 in cardiomyocytes (50). A positive association has been observed between serum S100A8/A9 levels and neutrophil counts in patients with Acute myocardial infarction (AMI) who are under dynamic monitoring. Research has validated that blood neutrophils are the sole cell population that significantly influences the levels of circulating S100A8/A9 in humans (13). Double immunostaining analysis of infarcted hearts from AMI patients revealed that the S100A8/A9 complex was primarily co-localized with neutrophils during the early acute phase and with macrophages in the subacute phase. Sreejit using cell sorting and flow cytometry to isolate leukocytes from infarcted hearts of mice, identified neutrophils as the principal source of S100A8/A9 (51). The infiltration of neutrophils and macrophages into the infarcted myocardium was found to be the main contributor to the elevated levels of S100A8/A9 in myocardial tissue following AMI (52). In response to myocardial infarction, a surge of neutrophils expressing the alert protein S100A8/A9 rapidly infiltrates the ischemic myocardium. S100A8/A9 then interacts with TLR4 on circulating neutrophils, triggering the formation of nucleotide-binding oligomerization domain-like receptor protein 3 (NLRP3) inflammatory vesicles and promoting IL-1 secretion in a mouse model of MI (53). The concept that S100A8/A9 is an upstream regulator of MI-induced granulopoiesis is supported by a mouse model with targeted disruption of S100A9, where the deletion of the S100A9 gene or pharmacological inhibition of S100A8/A9 by ABR-215757 markedly constrained granulopoiesis, reducing the number of circulating and cardiac neutrophils and monocytes (54, 55). The binding of S100A8/A9 to TLR4 leads to the upregulation of pro-inflammatory cytokine production by inducing the nuclear translocation of NF-κB via the Toll/interleukin-1 receptor (TIR) domain adapter, either through the interferon-β-dependent pathway or the MyD88-dependent pathway, thereby amplifying the inflammatory response (56). S100A8/A9 exerts a harmful influence during the initial phase of myocardial infarction (MI), and interventions aimed at inhibiting S100A8/A9 can be advantageous for MI patients. The S100A8/A9 complex interacts with the monocyte differentiation antigen CD69, leading to the formation of a CD69-S100A8/A9 complex (29). This association downregulates signaling and transcription by enhancing the expression of cytokine signaling repressor 3 (SOCS3) and inhibiting signaling and transcription activator 3 (STAT3) signaling, while promoting the differentiation of regulatory T cells (Tregs) (57). This mechanism may induce immunosuppression in various immune cells, preventing an overactive immune response. However, overzealous inhibition of S100A8/A9 could negate this beneficial effect during the MI repair phase. Prolonged inhibition of S100A9 in ischemic mice with ABR-238901 led to progressive left ventricular remodeling and a decline in cardiac function, indicating that S100A8/A9 may have additional cardioprotective roles during the recovery phase by promoting a shift in macrophage polarization towards a reparative phenotype (4, 58). These findings advocate for a tailored therapeutic approach, suggesting short-term anti-S100A9 blockade during the early inflammatory phase post-MI, while long-term blockade could impair cardiac function recovery during the repair phase, underscoring the need for precise therapy duration determination (59).

Research has identified that female individuals with high serum S100A8/A9 protein concentrations are at a 3.8-fold greater risk for adverse vascular outcomes (60). S100A8/A9 has been recognized as a new predictor for myocardial infarction, with incremental increases in S100A8/A9 protein levels associated with a proportional rise in the risk of subsequent cardiovascular events (24). Platelet mRNA profiling has demonstrated that S100A9 mRNA is significantly elevated in ST-segment elevation myocardial infarction(STEMI) patients compared to those with stable coronary artery disease. Additionally, serum S100A8/A9 concentrations are higher in AMI patients, particularly in those with cardiac rupture (61). Katashima et al. have reported on the dynamic monitoring of S100A8/A9 levels in patients with acute-phase AMI and Unstable angina pectoris(UAP), noting that initial levels are lower in AMI patients than in those with UAP, with a significant increase in AMI patients by 3–5 days post-event. S100A8/A9 levels are also higher in patients with ischemic injury compared to those with UAP, peaking 3–5 days after the ischemic event and remaining elevated for an extended period (51). A nested case-control study on ACS patients undergoing intensive lipid-lowering therapy for 30 days post-acute cardiovascular event identified elevated S100A8/A9 in those with MI or cardiovascular death. Nonetheless, S100A8/A9 has a limited diagnostic utility, with a sensitivity of 28% for myocardial infarction in patients presenting with non-traumatic chest pain, and it does not enhance the diagnostic accuracy provided by cardiac troponin (10).

### S100A8/A9 exacerbates ischemia/reperfusion (I/R) injury

Currently, an effective and standard treatment for STEMI is the reperfusion strategy (62). Reperfusion protects the ischemic heart from myocardial necrosis, but it also triggers a series of cascading responses that exacerbate and prolong post-ischemic injury (63). Inflammatory responses, oxidative stress (59), and mitochondrial dysfunction are important pathophysiological phenomena leading to alterations in cardiac structure and function after I/R injury (64). Du et al. found that S100A8/A9 was continuously expressed in mouse hearts during the early stages of myocardial ischemia/reperfusion injury (MI/RI) by time-series transcriptomics analysis, peaked at 6 h after reperfusion, and returned to baseline levels on day 7, thus clarifying that S100A8/A9 is an early mediator of I/R injury and is rapidly elevated in the early stages of MI/RI (65). Experimental Validation Findings S100A8/A9 was confirmed to be a key initiator molecule of I/R by S100A9 knockdown and overexpression experiments in animals, and in mice with a total deficiency of S100A9 after MI/RI, infarct size was significantly reduced, cardiac contractile function was improved, CM death was significantly reduced, and myocardial fibrosis was attenuated, suggesting that S100A9 increases the degree of myocardial ischemia-reperfusion injury (39, 43). Meanwhile mechanism studies revealed that S100A8/A9 down-regulated the gene expression of mitochondrial complex I subunit NDUFs by inhibiting the TLR4/ERK-mediated PGC-1α/NRF1 signaling pathway, which in turn inhibited the mitochondrial complex I function, causing mitochondrial dysfunction, leading to cardiomyocyte death, and promoting the development of MI/RI (66).

To fully understand the mechanism of action of S100A8/A9 in I/R, it is also important to clarify the cellular origin of S100A8/A9 in reperfusion injury. Experimental studies revealed that cardiac S100A8/A9 expression was significantly reduced in CXCR2-KO mice, further confirming that CXCR^2+^ neutrophils are the main source of S100A8/A9 secretion, which is related to the dynamic changes of CXCR2, and that cardiac neutrophil infiltration during I/R is consistent with the S100A8/A9 expression pattern. Chemokine (C-X-C motif) ligand 1 (CXCL1), a chemokine specific for CXCR2, is responsible for recruiting neutrophils expressing the chemokine receptor CXCR2 to the inflammatory microenvironment (67). In addition, mice treated with S100A9 neutralizing antibody (nAb) can observe a significant reduction in infarcted areas, increased cardiac function, and reduced myocardial fibrosis after I/R. Therefore, short-term blockade of S100A9 after I/R can effectively improve cardiac function in mice. Meanwhile, elevated serum S100A8/A9 levels 1 day after percutaneous coronary angioplasty (PCI) were found to be significantly associated with long-term adverse cardiovascular events in patients with acute myocardial infarction in the clinic (68). Targeting the signaling pathway initiated by S100A8/A9 may be a novel intervention for the treatment of MI/RI. At present, Du Jie's team has comprehensively carried out the work of early warning and intervention of S100A8/A9 after PCI for acute myocardial infarction: developed S100A8/A9 detection kit and monoclonal therapeutic antibody; and jointly carried out clinical research of “Early Warning of S100A8/A9 after PCI for Acute Myocardial Infarction”. Clinical research on S100A8/A9 in early warning after PCI for acute myocardial infarction (69).

### Inhibition of S100a8/A9 slows HF development

S100A8/A9 proteins are implicated in the advancement of HF by aggravating cardiac injury through the induction of localized inflammation (70). These proteins initiate the NF-κB signaling cascade by interacting with RAGE and TLR4 on the cell membrane, which in turn activates pro-inflammatory responses in cardiomyocytes, fibroblasts, and endothelial cells of cardiac tissue, thereby intensifying HF. Myocardial hypertrophy is a pivotal aspect of HF, and S100A8/A9 may be influenced by diverse physiological conditions, leading to varied responses in the pathogenesis of myocardial hypertrophy (40). While typically low in cardiomyocytes, S100A8 expression increases in response to thyroid hormone treatment in neonatal rat cardiomyocytes (71), contributing to cardiac hypertrophy via the MyD88/NF-κB pathway (72). Conversely, S100A8/A9 mRNA and protein levels rise when cardiomyocyte hypertrophy induced by norepinephrine ceases (73), potentially mitigating hypertrophy and remodeling by reducing calmodulin neuralphosphatase-nfatc3 activation (74). The mechanisms by which endogenous S100A8/A9 proteins, stimulated by different cues, differentially influence cardiomyocyte hypertrophic responses remain unclear. However, recombinant S100A8 protein application induces inflammatory responses in human pluripotent stem cell-derived cardiomyocytes, impacting calcium transport and electrophysiological properties. CD11b+Gr1+ neutrophil-derived S100A8/A9 proteins participate in angiotensin II-induced cardiac inflammation and fibrosis, activating NF-κB signaling in cardiac fibroblasts via RAGE and amplifying chemokine and cytokine production, thus intensifying the inflammatory response and cardiac remodeling that characterize HF progression (27).

Suppression of S100A8/A9 protein expression has been shown to exhibit significant anti-inflammatory and protective effects against myocardial hypertrophy and fibrosis across various tissues, thereby mitigating the progression of heart failure (HF). In a study involving angiotensin II-infused mice, the administration of a S100A9 antibody to neutrophils was observed to protect against myocardial hypertrophy and fibrosis. This treatment also reduced the infiltration of immune cells (CD45+ leukocytes, CD45+CD11b+ monocytes, and Gr1+ neutrophils) into myocardial tissues and curtailed NF-κB-dependent proinflammatory and profibrotic gene expression (75). In rats subjected to coronary ligation, the administration of the S100A8/A9 inhibitor ABR-215757 (5 mg/kg, daily for 5 days) led to a notable decrease in fibrotic areas surrounding the myocardial infarction margins and a reduction in NF-κB p65 protein levels (69). ABR-215757 has also demonstrated protective effects against tissue fibrosis in other experimental models (76). Concurrently, researchers have developed specific antibodies and vaccines targeting the mechanotransduction of S100A8/A9 proteins within the immune system, as well as compounds that block the interaction between S100A8/A9 and TLR4 and RAGE (35). Animal studies have confirmed that inhibiting S100A8/A9 protein expression has a beneficial anti-inflammatory effect, offering a promising therapeutic target for managing the inflammatory response in HF (77).

Experimental investigations have detected a variety of immune cells, such as neutrophils, T lymphocytes, macrophages derived from monocytes, and NK cells, in the cardiac tissues of HF patients who do not exhibit significant myocardial injury, viral infection, or known immune system anomalies (78), Neutrophils, especially, have been shown to contribute to the exacerbation of HF based on both clinical and animal research (79), and in HF with preserved ejection fraction, an elevated neutrophil count is a predictor of a poor outcome (80). The detection of high neutrophil expression in myocardial biopsies from HF patients with preserved ejection fraction and in HF model rats points to intense cardiac inflammation during the disease's progression (81). The role of S100A8/A9 proteins as neutrophil mediators in the promotion of chronic cardiac injury and inflammation is not yet fully elucidated (82). Transcription profiling has demonstrated increased S100A8/A9 gene expression in neutrophils from HF patients with preserved ejection fraction, indicating a connection between neutrophil activation and systemic inflammation, as well as left ventricular diastolic dysfunction in these patients (14). Proteomic analysis of platelets from HF patients with preserved ejection fraction has identified S100A8 protein in platelets and its increased levels in plasma (83). Moreover, in elderly HF patients, high S100A8/A9 protein concentrations have been positively correlated with the levels of the inflammatory cytokines Interleukin-6 (IL-6) and Interleukin-8 (IL-8). Collectively, these observations imply that S100A8/A9 proteins may function as biomarkers in the evolution of HF (84).

## Conclusions

Increasingly, research suggests that the concentrations of S100A8/A9 proteins in the bloodstream could be pivotal prognostic markers for negative cardiovascular outcomes in patients with acute and chronic heart failure, myocarditis, and thrombosis. Despite the intricate regulatory mechanisms of S100A8/A9, which involve complex transcriptional and post-translational processes and result in varied biological functions, the progress in developing potential therapeutics, such as humanized vaccines, antibodies, and inhibitors against S100A8/A9, is encouraging. These experimental findings have been successfully adapted to establish safe dosages for various immunoinflammatory diseases in clinical practice. Looking ahead, with the advancement of research technologies, the S100A8/A9 protein is expected to become a new focus for the diagnosis and treatment of cardiovascular diseases, potentially revolutionizing clinical approaches to diagnosis and therapy.
